# Increased production of ganoderic acids by overexpression of homologous farnesyl diphosphate synthase and kinetic modeling of ganoderic acid production in *Ganoderma lucidum*

**DOI:** 10.1186/s12934-019-1164-3

**Published:** 2019-06-28

**Authors:** Yu Fei, Na Li, De-Huai Zhang, Jun-Wei Xu

**Affiliations:** 1 School of Statistics and Mathematics, Yunnan University of Finance and Economics, Kunming, 650221 China; 2 Faculty of Life Science and Technology, Kunming University of Science and Technology, Kunming, 650500 China; 3 Faculty of Science, Kunming University of Science and Technology, Kunming, 650500 China

**Keywords:** *Ganoderma lucidum*, Ganoderic acid, Farnesyl diphosphate synthase, Genetic engineering, Modeling, Production kinetics

## Abstract

**Background:**

Ganoderic acids (GAs), derived from the medicinal mushroom *Ganoderma lucidum*, possess anticancer and other important pharmacological activities. To improve production of GAs, a homologous farnesyl diphosphate synthase (FPS) gene was overexpressed in *G. lucidum*. Moreover, the influence of FPS gene overexpression on GA production was investigated by developing the corresponding mathematical models.

**Results:**

The maximum levels of total GAs and individual GAs (GA-T, GA-S, and GA-Me) in the transgenic strain were 2.76 mg/100 mg dry weight (DW), 41 ± 2, 21 ± 5, and 28 ± 1 μg/100 mg DW, respectively, which were increased by 2.28-, 2.27-, 2.62-, and 2.80-folds compared with those in the control. Transcription levels of squalene synthase (SQS) and lanosterol synthase (LS) genes during GA biosynthesis were upregulated by 2.28- and 1.73-folds, respectively, in the transgenic *G. lucidum*. In addition, the developed unstructured models had a satisfactory fit for the process of GA production in submerged cultures of *G. lucidum*. Analysis of the kinetic process showed that FPS gene overexpression had a stronger positive impact on GA production compared with its influence on cell growth. Also, FPS gene overexpression led to a higher non-growth-associated-constant *β* (1.151) over the growth-associated-constant *α* (0.026) in the developed models.

**Conclusions:**

FPS gene overexpression is an effective strategy to improve the production of GAs in *G. lucidum*. The developed mathematical models are useful for developing a better GA production process in future large-scale bioreactors.

**Electronic supplementary material:**

The online version of this article (10.1186/s12934-019-1164-3) contains supplementary material, which is available to authorized users.

## Background

Mushrooms are rich sources of biologically active compounds and cosmetic ingredients [1, 2]. *Ganoderma lucidum*, a traditional medicinal mushroom, has been used to treat and prevent various diseases for two millennia in Asian countries [3, 4]. The market size of *Ganoderma* products is estimated to be worth over US $2.5 billion (Bishop et al. [5]). Ganoderic acids (GAs), a type of tetracyclic triterpenoid, are one of the major ingredients of *G. lucidum* and have diverse pharmacological activities, including antitumor, anti-metastasis, anti-HIV, antiviral, hepatoprotective, hypocholesterolemic antioxidant, and antiaging effects [6–8].

**Fig. 1:**
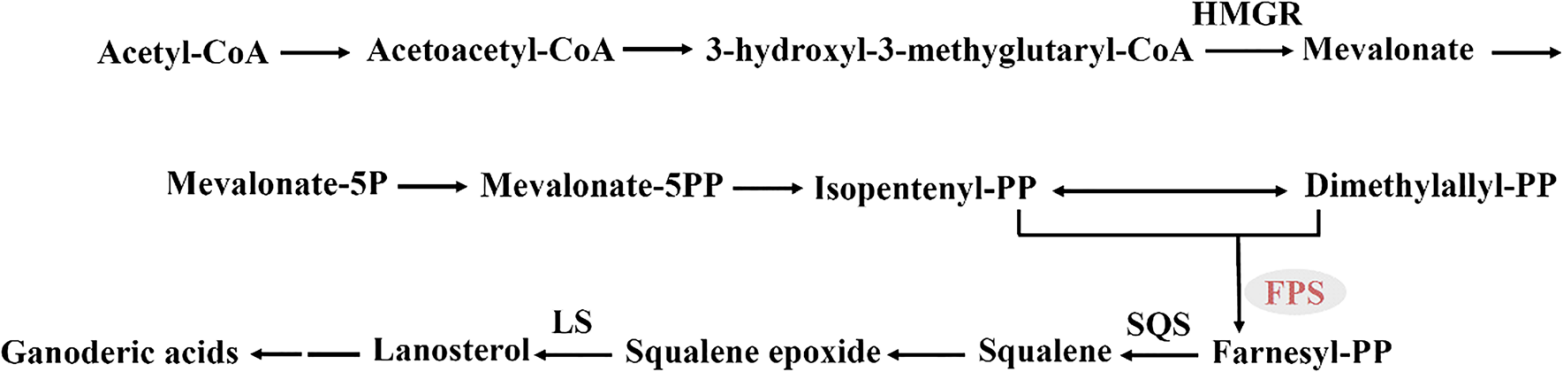
The biosynthetic pathway of ganoderic acids

GAs are biosynthesized via the mevalonate/isoprenoid (MVA) pathway from acetyl-coenzyme A to produce lanosterol in *Ganoderma* species (Fig. 1) [8–10]. Further biosynthetic steps to form GAs probably include a series of complex oxidation, reduction, hydroxylation, and acylation reactions [8, 11]. Some genes involved in GA biosynthetic pathways have been characterized in *G. lucidum* [9, 12–15]: 3-hydroxy-3-methyglutaryl coenzyme A reductase (HMGR), farnesyl-diphosphate synthase (FPS), squalene synthase (SQS), and lanosterol synthase (LS) have been identified as key enzymes involved in GA biosynthesis [9, 16–19].

Despite their important biological functions, the low production of GAs is a bottleneck for clinical trials and commercial applications [7, 20]. At present, GAs are mainly obtained from fruiting bodies and mycelia of *G. lucidum*. Compared to fruiting body cultures, a submerged culture is a promising alternative for the production of triterpenoids, as it is easy to control the product quality and is cost effective [21, 22]. Many attempts, such as by manipulating fermentation conditions [6, 23], developing bioprocessing and elicitor strategies [24–29], and metabolic engineering [10, 14, 15, 17, 18, 30, 31], have been conducted to increase the production of GAs by fermentation of mycelia.

Farnesyl diphosphate synthase (FPS) catalyzes the condensation reactions of dimethylallyl diphosphate with two units of isopentenyl pyrophosphate to produce farnesyl pyrophosphate, which is located at the branch point in the MVA pathway for several terpenes, like sesquiterpenes, sterols, and triterpenes [32, 33]. In hairy roots of *Artemisia annua*, overexpression of the FPS gene from *Gossypium arboretum* increases the accumulation of the sesquiterpene artemisinin [32]. FPS gene overexpression leads to significant increases in sterol and carotenoids in *Nicotiana tabacum* [34]. In *Panax ginseng* adventitious roots, overexpression of the FPS gene enhances the levels of triterpene ginsenosides [35]. The *G. lucidum* FPS gene has been cloned and characterized, and its transcription level exhibits a positive correlation with the triterpene content during fruiting body development [36]. These results indicate that FPS plays an important role in the control of triterpene biosynthesis. In *G. lucidum*, enhanced levels of GAs have been achieved by overexpression of some structural genes involved in the upstream biosynthetic pathway [14, 15, 17, 18]. However, the genetic manipulation of the FPS gene, which may regulate GA biosynthesis, has yet to be performed.

Mathematical models are important engineering tools, and they are useful for understanding bioprocess behavior, control, and optimization. Unstructured models can describe the fermentation profiles of products such as polysaccharides, gibberellic acid, triterpenoids, and flavonoids with satisfactory accuracy [24, 37–39]. Previously, Tang and Zhong described an unstructured mathematical model for GA biosynthesis by *G. lucidum* in liquid static culture conditions [40]. Feng et al. proposed an unstructured model for triterpenoid production in submerged fermentation of *G. lucidum* G0119 [24]. However, all those models are about fermentation of wild-type (WT) *G. lucidum*. Kinetic models of GA production by genetically modified strains remain to be produced. The development of such mathematical models could improve our understanding of the effects of FPS gene overexpression on the kinetics of cell growth, GA production, and sugar consumption.

In this study, the effect of FPS gene overexpression on the level of GAs and the transcription levels of key enzymes (HMGR, SQS, and LS) of the GA biosynthetic pathway were investigated in submerged cultivation of *G. lucidum*. Moreover, the impact of FPS gene overexpression on the production of GAs by *G. lucidum* was also analyzed by developing corresponding mathematical models. This study sheds light on GA biosynthesis regulation and will help us to develop efficient production of GAs on a larger scale.

## Results

### Generation of homologous FPS-overexpressing *G. lucidum*

**Fig. 2:**
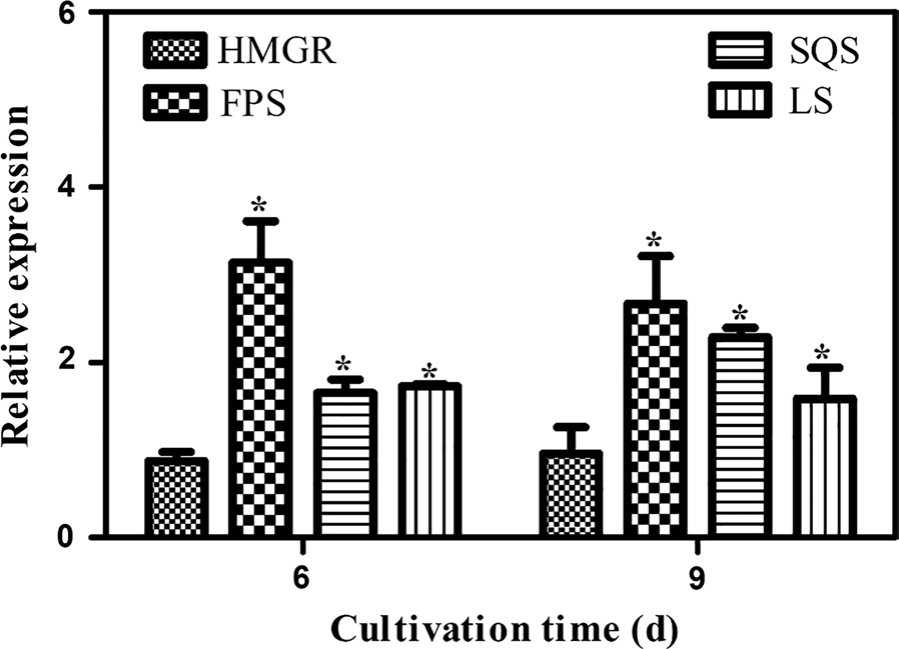
Transcriptional levels of GA biosynthetic genes in the WT and the FPS transgenic strain. Expression of samples from the WT strain is defined as 1.0, and expression levels in the FPS strain are displayed as fold increases over the reference sample. *d* days. * indicates statistical significance (*P* < 0.05) compared to the WT strain

The *G. lucidum* FPS gene was amplified by genomic PCR with the primers FPS-*Nhe*I-F and FPS-*Sma*I-R. To overexpress the FPS gene, the plasmid pJW-EXP-FPS (Additional file 1: Fig. S1) was constructed and introduced into *G. lucidum* using the reported genetic transformation method [10, 14, 41]. The expression of the FPS gene was driven by the glyceraldehyde-3-phosphate dehydrogenase gene promoter in the pJW-EXP-FPS plasmid. Transformants were screened on selective CYM medium with 2 μg/mL carboxin after five rounds of culture on carboxin-free medium. Those transformants maintaining antibiotic resistance were further confirmed via genomic PCR analysis, followed by sequencing of the obtained PCR products. A 1.11 kb amplification band, representing the fused fragment of the glyceraldehyde-3-phosphate dehydrogenase gene promoter and FPS gene, was detected in the lanes of the FPS transformant and the pJW-EXP-FPS plasmid, whereas no corresponding band was detected in the lane of WT *G. lucidum* (Additional file 1: Fig. S1). In addition, no differences were observed in morphology between the FPS transformant and WT strains (data not shown). The results of qRT-PCR analysis revealed that FPS gene overexpression induced accumulation of its transcripts in *G. lucidum* (Fig. 2). The transcript levels of the FPS gene in the transformant under submerged culture conditions were 3.12 and 2.71 times higher than those of the WT strain on days 6 and 9, respectively. Altogether, these results demonstrated that FPS-overexpressing *G. lucidum* was generated successfully, and that it gives rise to high transcription levels of the FPS gene.

### Increased GA biosynthesis in *G. lucidum* overexpressing the homologous FPS gene

To determine whether FPS gene overexpression contributes to the accumulation of GAs in *G. lucidum*, the contents of total GAs and individual GAs in the WT strain and three *G. lucidum* transformants (namely, FPS1, FPS2, and FPS3) were analyzed in submerged cultivation conditions. Table 1 shows that the total GA levels in FPS1, FPS2, and FPS3 strains were increased to 2.28, 2.15, 2.12 times that of the WT strain (1.21 mg/100 mg DW). GA-Mk, GA-T, GA-S, and GA-Me were detected as major GA components from *G. lucidum* mycelia [23]. The levels of individual GAs were also measured in different G*. lucidum* strains. The contents of GA-Mk, GA-T, GA-S, and GA-Me in the FPS-1 transformant were 4 ± 1, 41 ± 2, 21 ± 5, and 28 ± 1 μg/100 mg DW, respectively, which were increased by 2-, 2.23-, 2.74-, and 2.85-fold compared with those of the untransformed WT strain. These results showed that overexpression of the homologous FPS gene increased GA accumulation in *G. lucidum*.

**Table 1:** Content of total GAs, GA-Mk, GA-T, GA-S and GA-Me in different cell lines

Cell line	Biomass (g/L)	Total GA content (mg/100 mg DW)	Individual GA content (μg/100 mg DW)			
			Mk	T	S	Me
WT	8.26 ± 0.34	1.21 + 0.09	2 ± 0	18 ± 1	8 ± 1	10 ± 1
FPS1	8.13 ± 0.21	2.76 + 0.38*	4 ± 1*	41 ± 2*	21 ± 5*	28 ± 1*
FPS2	8.31 ± 0.27	2.61 + 0.21*	3 ± 0*	44 ± 2*	22 ± 4*	30 ± 2*
FPS3	8.27 ± 0.31	2.57 + 0.31*	4 ± 0*	42 ± 2*	21 ± 4*	27 ± 1*

* Significantly different from value for WT (*P* < 0.05)

To investigate the regulatory role of the FPS gene in the GA biosynthetic pathway, the mRNA accumulations of HMGR, SQS, and LS genes were examined in the FPS transformant and WT strains by qRT-PCR. Samples were measured on days 6 and 9 in accordance with our preliminary experiments. Figure 2 shows that overexpression of the FPS gene significantly improved the transcription levels of SQS and LS genes. The maximum transcription levels of SQS and LS genes in the FPS transformant were 2.28- and 1.73- fold higher than those in the WT strain. However, no differences were detected in transcription levels of the HMGR gene between the FPS transformant and control strain. From the above information, the transformant FPS1 and the WT strain were selected to further study the kinetic profiles of cell growth, residual sugar, and total GA production.

### Mathematical model development for production kinetic profiles in *G. lucidum*

**Fig. 3:**
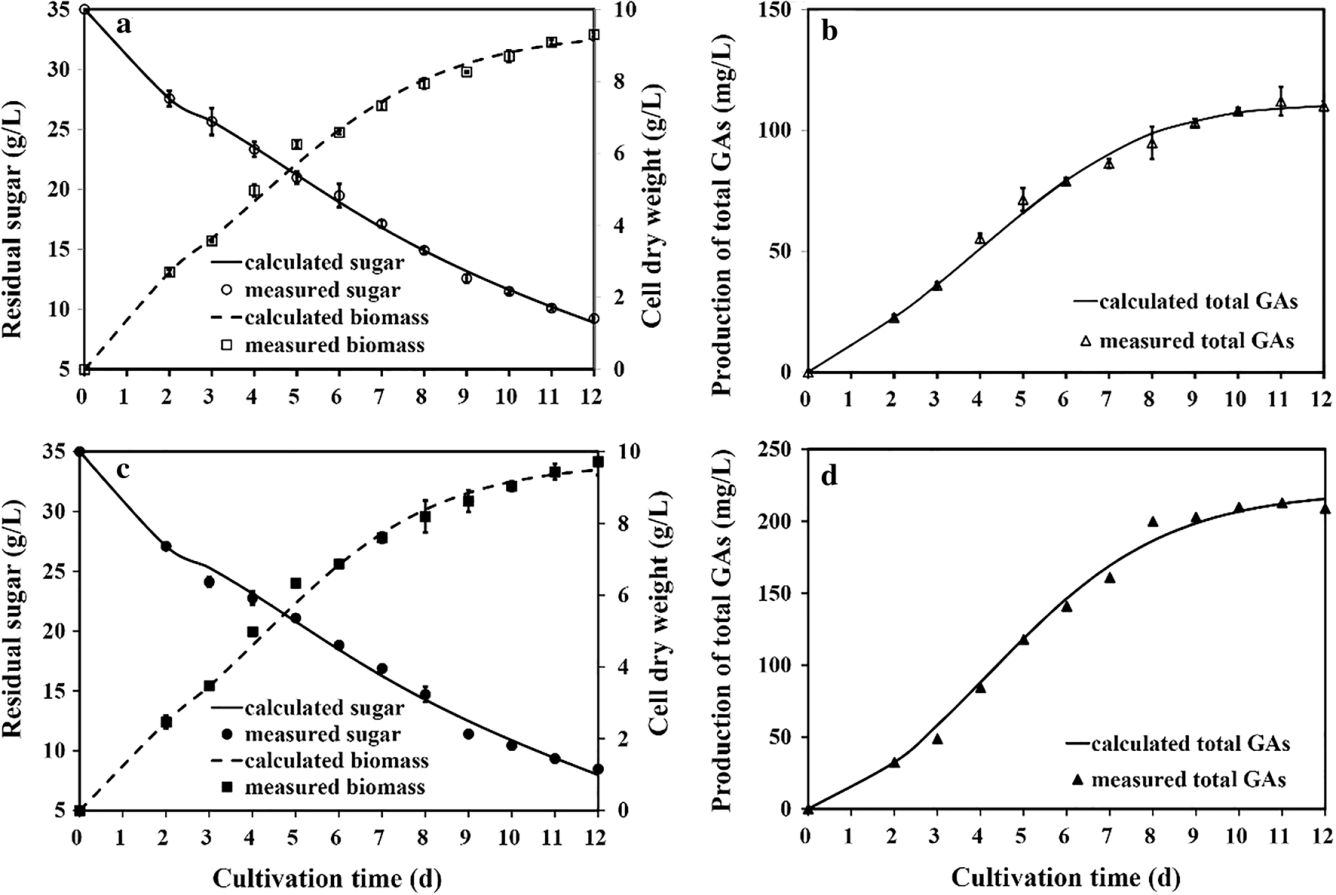
Simulation of GA fermentation process for biomass, residual sugar, and GA production in submerged cultivation of the WT (**a, b**) and the *G. lucidum* FPS-overexpressing strain (**c**, **d**)

The time-course of cell growth, sugar utilization, and GA production by *G. lucidum* under submerged culture conditions is shown in Fig. 3a, b. A maximum biomass of 9.30 g/L was obtained after 12 days of fermentation when the residual sugar concentration was decreased to 9.25 g/L. The total GA production increased slowly with cell growth and reached its maximum of 0.112 g/L at day 12.

These experimental data were used to determine the kinetic parameters and develop mathematical models to describe cell growth, GA production, and sugar consumption. After fitting the data for cell growth (DW, g/L), GA production (g/L), and sugar consumption (g/L) to Eqs. (1)–(3) using 1st Opt statistical analysis software, the model parameters were calculated to describe the responses as a function of time. The experimental data and model simulation of *G. lucidum* culture are shown in Fig. 3a, b. The calculated parameters and the correlation coefficients (*R*^2^) are summarized in Table 2. The maximum specific growth rate (*μ*_max_) and maximum biomass concentration (*X*_max_) were 0.45 (day^−1^) and 9.41 (g/L), respectively. The other parameters derived from the model were as follows: *α* = 0.015 (g GAs g^−1^ DW), *β* = 0 (g GAs/g^−1^ DW day^−1^), *Y*_*X*/*S*_ = 0.63 (g DW g^−1^), *M*_s_ = 0.12 (g sugar g^−1^ DW day^−1^). As evident from the results shown in Table 2, the correlation coefficient (R^2^) for the kinetic model was 0.99, which showed that the kinetic model gave a satisfactory fit to the experimental data. Thus, the developed mathematical models accurately described the process of GA production in submerged culture conditions and were suitable for predicting experimental results.

**Table 2:** Estimated parameter values of kinetic model equations

Parameter	WT	FPS
Biomass		
*μ*_max_ (day^−1^)	0.45	0.49
*X*_max_ (g L^−1^)	9.41	9.72
*V*_max_ (g day^−1^)	1.05	1.18
Sugar		
*Y*_*X*/*S*_ (g g^−1^)	0.63	0. 70
*M*_s_ (g g^−1^ day^−1^)	0.12	0.13
GA formation		
*α* (g g^−1^)	0.015	0.026
*β* (g g^−1^ day^−1^)	0	1.151
*P*_max_ (g L^−1^)	0.112	0.209
Productivity (g L^−1^ day^−1^)	0.009	0.018
*F* value	52.9	51.8
*R*^2^	0.99	0.99

WT, wide-type strain; FPS, the FPS gene overexpressed strain

### The effect of overexpression of homologous FPS on GA production through the mathematical models

To study the effect of FPS gene overexpression on GA biosynthesis, the production of total GAs was assessed in submerged cultivation of the FPS transformant. A comparison between the experimental data and simulated values was made to test the mathematical models (Fig. 3c, d). All simulated values showed a high nonlinear coefficient with the observed values (*R*^2^ = 0.99). Fitness of the experimental data with respect to *F* values in the models was statistically satisfactory (Fig. 3c, d and Table 2). Therefore, the data fitted the mathematical models well according to the statistical results (Table 2). These results suggested that these mathematical models can describe GA production by the FPS transformant in submerged culture conditions.

Figure 3c indicates that the FPS transformant grows at the same rate as the WT strain [18]. The maximum biomass value (*X*_max_) of the FPS transformant was 9.72 g/L, showing a slight increase compared with that of the WT strain (9.41 g/L). Table 2 shows that the values of *μ*_max_ (day^−1^) and *V*_max_ (g day^−1^) from the FPS transformant only had 8% and 12% increases in comparison to those from the WT strain, respectively. These results suggest that FPS gene overexpression does not significantly affect cell growth during GA production. Analysis of sugar utilization indicated that sugar consumption patterns showed a continuous decline in both the FPS transformant and WT strains during fermentation, which is consistent with the cell growth profiles. The *M*_s_ and *Y*_*X*/*S*_ in the FPS transformant were 0.13 (g sugar g^−1^ DW day^−1^) and 0.70 (g DW g^−1^ sugar), respectively, which are comparable to those obtained in the WT strain. These results indicated that overexpression of the FPS gene had no significant impact on sugar consumption. The final maximum production of GAs reached 0.209 g/L in the FPS transformant, which was higher than that in the control (0.171 g/L). FPS overexpression resulted in an improvement in productivity, with a value of 0.018 g day^−1^, twofold higher than that in the control (0.009 g day^−1^), which improved the process efficiency. The growth-associated parameter *α* and non-growth-associated parameter *β* for the FPS transformant were 0.026 (g GAs g^−1^ CDW) and 1.151 (g GAs g^−1^ CDW day^−1^) in submerged culture conditions, respectively. These results suggested that FPS overexpression has a positive impact on GA formation in terms of production and productivity.

## Discussion

Although the FPS gene has been characterized from the basidiomycete *G. lucidum*, there have been no reports on the manipulation of GA biosynthesis based on the expression of the FPS gene. In this study, the homologous FPS gene was chosen for overexpression in order to increase production of GAs in *G. lucidum*. Moreover, an unstructured mathematical model was developed to investigate the effect of FPS gene overexpression on the production of total GAs by *G. lucidum*.

The FPS gene can be successfully introduced into *G. lucidum* as confirmed by genomic PCR and qRT-PCR analysis. FPS gene overexpression resulted in improvement of total and individual GA contents. The obtained contents of GAs in the transgenic strain were comparable to those in *G. lingzhi* overexpressing the squalene epoxidase gene [14]. The results indicated that FPS plays a regulatory role in GA biosynthesis. This report is in agreement with previous findings in *P. ginseng* and *N. tabacum*. Overexpression of the *Centella asiatica* FPS gene improved the contents of seven total ginsenosides in *P. ginseng* [35]. The accumulation of tetraterpene carotenoids and sterols has also been increased by heterologous expression of the FPS gene from *Saccharomyces cerevisiae* in tobacco [34]. Overexpression of the FPS gene may increase the metabolic flux of intermediates towards the triterpene biosynthetic pathway, thus leading to the increased accumulation of GAs in *G. lucidum*. The accumulation of GAs coincided with the increased transcription levels of the FPS gene, suggesting that the increased transcription level may, at least in part, be responsible for the elevation in GAs in the transgenic strain. In previous studies, a positive correlation between the transcription level of the FPS gene and the content of total triterpenoids during development was reported in *G. lucidum* and *Poria cocos* [36, 42]. Overexpression of the FPS gene induced the upregulation of SQS and LS genes rather than the HMGR gene in the transgenic strain, which is the same as previous reports showing that the manipulation of downstream genes in triterpene biosynthesis does not significantly affect the transcription levels of upstream genes [15, 18, 43].

A correlation with high significance (*R*^2^) was obtained between experimental results (for biomass, GA production, and sugar consumption) and model parameters with significant statistical fitness (*F* test) values. Calculated and observed time-courses exhibited good agreement in both the WT and FPS transgenic strains in this work. The developed mathematical models fit the experimental data well, and these models provide new information on the effects of FPS gene overexpression on GA production by *G. lucidum* with respect to the kinetic process. This information will be useful for optimizing and scaling-up of GA production in future bioreactors. Analysis of GA production showed that FPS gene overexpression had a stronger positive impact on GA production than on cell growth and consumption of sugar. Overexpression of the FPS gene led to the enhancement of GA accumulation and productivity. The growth coefficient *Y*_*X*/*S*_ (0.63 and 0.70 for the WT and FPS strains, respectively) obtained was comparable to that (0.78 g g^−1^) reported by Tang and Zhong [40]. The maximum specific growth rate of 0.448–0.483 day^−1^ in liquid submerged culture of *G. lucidum* was higher than the *μ*_max_ of 0.23 day^−1^ in a previous study that modeled the growth profiles in liquid static culture conditions [40]. The longer fermentation time in the liquid static culture model may be responsible for the difference in the specific growth rate. The obtained higher *α* value over *β* (*α* = 0.015 g GAs g^−1^ DW and *β* = 0 g GAs g^−1^ DW day^−1^) in the WT strain indicated that a growth-associated factor plays an important role in GA production. These results are consistent with previous reports in liquid-submerged cultivation of *G. lucidum* G0119 and in liquid static culture of *G. lucidum* [24, 40]. However, the much higher value of *β* over *α* (*α* = 0.026 g GAs g^−1^ DW and *β* = 1.151 g GAs g^−1^ DW day^−1^) in the transgenic strain suggested that GA production is more related to the obtained biomass than to the rate of cell growth. This may be due to the higher content of total GAs in *G. lucidum* overexpressing the FPS gene.

## Conclusions

Overexpression of the homologous FPS gene upregulated the transcription levels of SQS and LS genes, and increased production of GAs in a submerged *G. lucidum* culture. Moreover, empirical mathematical models were developed to explore the effect of FPS gene overexpression on GA production. The results indicated that FPS gene overexpression is an effective strategy to improve the production of GAs in *G. lucidum*. The developed mathematical models may be useful for developing a better GA production process in future large-scale bioreactors.

## Methods

### Strains and culture conditions

*Ganoderma lucidum* CGMCC 5.616 culture was purchased from the China General Microbiological Culture Collection Center. *Escherichia coli* strain JM109 is routinely grown in our laboratory. *G. lucidum* was cultured as described by Xu et al. [23]. Protoplasts of *G. lucidum* were regenerated in CYM media that contained 20 g/L glucose, 10 g/L maltose, 0.6 M mannitol, 2 g/L yeast extract, 2 g/L tryptone, 0.5 g/L MgSO_4_, 4.6 g/L KH_2_PO_4_, and 10 g/L agar.

### Vector construction and transformation

The FPS gene of *G. lucidum* (GenBank accession number: EU399544) was acquired by genomic PCR using primers FPS-*Nhe*I-F (5′-GCTAGCATGGCCGATGCAAAGGCTCAG-3′) and FPS-*Sma*I-R (5′-CCCGGGTCACTTCTGCCGCTTGTAGATCTTGTC-3′). The *G. lucidum* FPS gene was double-digested with *Nhe*I and *Sma*I, and then ligated into the pJW-EXP vector [41], forming pJW-EXP-FPS. *G. lucidum* protoplasts were transformed with 10 μg of pJW-EXP-FPS by polyethylene glycol-mediated genetic transformation as described previously [10, 41, 44]. Transformants were plated on CYM regeneration plates containing 2 μg carboxin/mL to isolate resistant clones. The genomic DNA of *G. lucidum* was extracted using the cetyltrimethylammonium bromide (CTAB) method. Stable transformants were confirmed by genomic PCR analysis using the primer pair GPD-FPS-F (5′-CGTAGCAATGCCAGGAAA-3′) and GPD-FPS-R (5′-TTGAGGATCTCGACGGAGT-3′) and sequencing.

### Gene expression analysis by quantitative real-time PCR (qRT-PCR)

Total RNAs of *G. lucidum* were extracted using TriZol Reagent (Invitrogen, Carlsbad, CA), treated with DNAase I (Fermentas, Canada), and then reverse-transcribed to cDNA using a Superscript RNAase H-First-strand synthesis kit (Invitrogen). The transcriptional levels of HMGR, FPS, SQS, and LS genes were determined by quantitative real-time PCR. Primers and PCR protocols used in the quantitative real-time PCR were described previously [17, 30]. The transcription levels of HMGR, FPS, SQS, and LS genes were normalized relative to the transcription level of the 18S rRNA gene. The transcription levels of samples from the wild-type (WT) strain were defined as 1.0, and the transcription levels in the transgenic strain are presented as fold changes with respect to these reference levels. Post-quantitative real-time PCR calculations were performed according to the 2^−ΔΔCt^ method.

### Analysis of biomass, total GAs and individual GAs

*Ganoderma lucidum* mycelia were collected during cultivation and were centrifuged at 10,000×*g* for 10 min, washed three times with water, and dried at 45 °C. Biomass was determined by the gravimetric method. The total GAs and individual GAs of mycelia were extracted and measured using methods described in our previous work [17, 23].

### Statistical analysis

Data are the averages of three independent sample measurements. The results were analyzed for statistical significance by Student’s *t* test. The differences were considered significant at *P* < 0.05 in a two-tailed analysis. All data are represented as mean ± standard deviation. The 1st Opt statistical analysis software (http://www.7d-soft.com) was used to fit the developed models and estimate the parameters based on universal global optimization (UGO) algorithms. 1st Opt can be used to preform nonlinear regression, curve fitting, and for the optimization of nonlinear model parameters [45].

### Mathematical model development

#### Mycelia growth

For mycelia growth, a logistic model was used to describe the growth of *G. lucidum*:

1 $$\frac{{{\text{d}}X}}{{{\text{d}}t}} = \mu_{\text{max} } \times \left( {1 - \frac{X}{{X_{\text{max} } }}} \right)$$

where $\frac{{{\text{d}}X}}{{{\text{d}}t}}$ is the rate of cell growth, *X* is the cell dry weight (g DW L^−1^), *μ*_max_ is the maximum specific growth rate (day^−1^), and *X*_max_ is the maximum cell dry weight (g DW L^−1^).

#### Production of total GAs

The kinetics of the formation of total GAs were simulated by the Luedeking–Piret model:

2 $$\frac{{{\text{d}}P}}{{{\text{d}}t}} = \alpha \frac{{{\text{d}}X}}{{{\text{d}}t}} + \beta X,$$

where $\frac{{{\text{d}}P}}{{{\text{d}}t}}$ is the GA formation rate, *P* is the production of total GAs (mg L^−1^), *α* is the constant for growth-associated kinetics of GA production (g GAs g^−1^ DW), and *β* is the non-growth-associated-constant (g GAs/g^−1^ DW day^−1^).

#### Sugar consumption

The sugar consumption equation is a Luedeking–Piret-like equation in which the amount of sugar used for GA production is assumed to be negligible:

3 $$- \frac{{{\text{d}}S}}{{{\text{d}}t}} = \frac{1}{{Y_{X/S} }}\frac{{{\text{d}}X}}{{{\text{d}}t}} + M_{\text{s}} X ,$$

where $- \frac{{{\text{d}}S}}{{{\text{d}}t}}$ is the consumption rate of sugar, *S* is the residual sugar concentration (g L^−1^), *Y*_*X*/*S*_ (g DW g^−1^ sugar) is the maximum cell growth coefficient, and *M*_s_ is the maintenance constant (g sugar g^−1^ DW day^−1^).

## Additional file

**Additional file 1: Figure S1.** (A) The expression vector pJW-EXP-FPS. (B) Identification and characterization of the FPF gene-overexpressing strain. Amplification pattern of genomic PCR obtained with primers for the fusion of the *gpd* promoter and FPS gene fragment from different strains.

## Data Availability

Identification and characterization of the FPS gene-overexpressing strain.

## Abbreviations

GA: ganoderic acid
FPS: farnesyl-diphosphate synthase
WT: wild-type
DW: dry cell weight
HMGR: 3-hydroxy-3-methylglutaryl coenzyme A
SQS: squalene synthase
LS: lanosterol synthase
CYM: complete yeast medium
*gpd*: glyceraldehyde-3-phosphate dehydrogenase gene
UGO: universal global optimization
