# Supplementary material for: Increased production of ganoderic acids by overexpression of homologous farnesyl diphosphate synthase and kinetic modeling of ganoderic acid production in Ganoderma lucidum
Source: Microb Cell Fact. 2019 Jun 28;18:115. doi: 10.1186/s12934-019-1164-3 (PMC6599323; doi:10.1186/s12934-019-1164-3)
Supplement: Supplementary file 1 — Additional file 1: Figure S1. (A) The expression vector pJW-EXP-FPS. (B) Identification and characterization of the FPF gene-overexpressing strain. Amplification pattern of genomic PCR obtained with primers for the fusion of the gpd promoter and FPS gene fragment from different strains. [file 12934_2019_1164_MOESM1_ESM.docx]

**Increased production of ganoderic acids by overexpression of homologous farnesyl diphosphate synthase and kinetic modeling of ganoderic acid production in *Ganoderma lucidum***

Yu Fei^1,a^, Na Li^1,a,c^, De-Huai Zhang^b^ Jun-Wei Xu^a,b,*^

a. School of Statistics and Mathematics, Yunnan University of Finance and Economics, Kunming, 650221, China

b. Faculty of Life Science and Technology, Kunming University of Science and Technology, Kunming, 650500, China

c. Faculty of Science, Kunming University of Science and Technology, Kunming, 650500, China

*, Corresponding author: Jun-Wei Xu (xjuwei@163.com; jwxu@kmust.edu.cn; Tel/Fax: +86-871-65920676)

1, Contributed equally to this work.


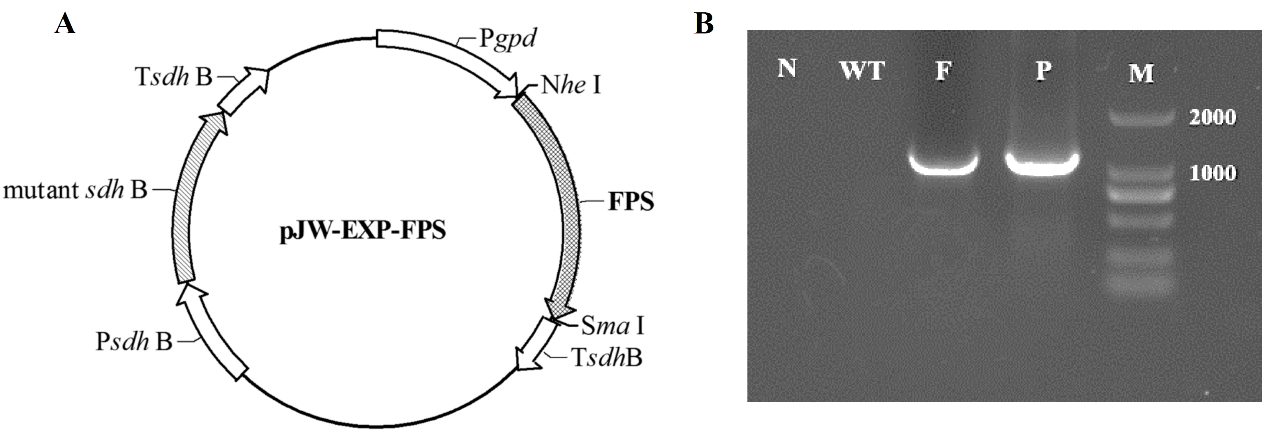


Figure S1 (A) The expression vector pJW-EXP-FPS. (B) Identification and characterization of the FPS gene-overexpressing strain. Amplification pattern of genomic PCR obtained with primers for the fusion of the *gpd* promoter and FPS gene fragment from different strains. Lane N, negative control; Lane WT, wild-type strain; Line F, the FPS gene-overexpressing strain; Lane P, pJW-EXP-FPS as a positive control; Lane M, DL 2000 DNA marker.
